# Combinations of Peptide-Protein Extracts from Native Probiotics Suppress the Growth of Multidrug-Resistant *Staphylococcus aureus* and *Citrobacter freundii* via Membrane Perturbation and Ultrastructural Changes

**DOI:** 10.3390/antibiotics11020154

**Published:** 2022-01-25

**Authors:** Gabriela N. Tenea, Evelyn Angamarca, Daniela Olmedo

**Affiliations:** Biofood and Nutraceutics Research and Development Group, Faculty of Engineering in Agricultural and Environmental Sciences, Technical University of the North, Av. 17 de Julio s-21. Barrio El Olivo, Ibarra 100150, Ecuador; elangamarcac@utn.edu.ec (E.A.); dolmedof@utn.edu.ec (D.O.)

**Keywords:** antimicrobials, *Staphylococcus aureus*, *Citrobacter freundii*, probiotics, membrane alteration, TEM, SEM, “fried egg” phenotype

## Abstract

The occurrence of multidrug-resistant pathogens in the food chain causes health problems in humans, thus, research for novel antimicrobials to combat their growth is of interest. This study evaluates the antimicrobial potential of several combinations of peptide-protein extracts (PCs) consisting of peptide extracts from three native probiotic strains, *Lactiplantibacillus plantarum* UTNGt2, *Lactococcus lactis* UTNGt28, and *L. plantarum* UTNGt21A, alone or in combination with EDTA (ethylenediaminetetraacetic acid) against multidrug-resistant *Staphylococcus aureus* ATCC1026 and *Citrobacter freundii* UTNB3Sm1. Based on the antimicrobial assay, among the 19 tested PCs, two (PC11 and PC17) produced a greater zone of inhibition against both pathogens in vitro. Time-killing assays indicated the rapid death of *S. aureus* after exposure to PC11 and PC17, while *C. freundii* was rapidly inhibited by PC11 and PC1 (UTNGt2 only), suggesting that the inhibitory action is pathogen and dose-dependent of a particular molecule present in the extract. A marginal inhibitory effect was observed when the peptides were combined with EDTA. Transmission electron microscopy (TEM) revealed the structural membrane damage of both target strains upon interaction with individual peptide extracts. Different degrees of cell deformation, condensed cytoplasm, membrane blebbing, and ghost cell formation with visibly broken cell walls were observed in *S. aureus*. Likewise, the separation of the cytoplasmic membrane from the outer membrane, ghost cells, along with ovoid and deformed cells with undulated cell walls were observed for *C. freundii*. Furthermore, scanning electronic microscopy (SEM) analysis revealed different wrinkled and deformed cells covered by debris. A leakage of aromatic molecules was detected for both pathogens, indicating that PCs disrupted the cell wall integrity, inducing cell death. Given their inhibitory action and capacity to induce damage of the cytoplasmic membrane, the selected PCs may serve to slow bacterial growth in vitro; further research is required to prove their efficiency ex vitro to battle against food poisoning and subsequent human infection.

## 1. Introduction

The diseases associated with multidrug-resistant microorganisms such as *Staphylococcus aureus, Salmonella* spp., *Clostridium perfringens, E. coli* spp., and other *Enterobacteriaceae,* remain a public health concern [1,2]. *S. aureus,* a Gram-positive and cocci-shaped bacterium, is a pathogen involved in nosocomial and community-acquired infections, including skin tissue infection and pneumonia [3]. Several strains exhibit a multidrug-resistant phenotype, survive, and grow in a wide range of environmental conditions, having been detected in vegetables, fruits, meat, and bakery products, which impose a high risk for human health [4]. *Citrobacter freundii*, a member of *Enterobacteriaceae,* is a facultatively anaerobic, motile, and Gram-negative bacillus, widely distributed in the environment and intestinal tracts of humans and animals, so this bacillus is regarded as an environmental contaminant or harmless colonizer [5]. *Citrobacter* ssp. are opportunistic human pathogens [6]. Very early research associates the presence of *C. freundii* with neonatal meningitis and bilateral brain abscesses [7]. Severe foodborne gastroenteritis outbreaks attributed to *C. freundii* have been previously reported [8,9,10,11]. The infection was attributed to the presence of *Citrobacter* in vegetables. Besides, *Citrobacter* spp. were found multidrug-resistant to β-lactams, quinolones, aminoglycosides, tetracyclines, sulphone amides [12,13]. Thus, the control or prevention of the growth of these pathogens in different food matrices is necessary. 

Worldwide, chemical preservatives are commonly used to prevent pathogen growth in foods, but their negative effects on organoleptic properties and human health are receiving growing attention [14]. The demand to replace the chemicals with natural substances produced by lactic acid bacteria (LAB) has expanded further investigation [7]. In the last decade, probiotic bacteria have been screened to produce antimicrobials [15]. Probiotics are unique bacteria that produce metabolites and antimicrobial peptides (AMP) with potential immunomodulatory and anti-inflammatory activities, thus offering several therapeutic benefits [16]. AMPs are widely used in the food industry as biopreservatives due to their ability to reduce microbial growth and control deterioration in many foods, improving their shelf life without compromising the quality, managing to satisfy consumer demand for safe and secure food [17]. Thus, AMPs are considered as an alternative to antibiotics against resistant strains [18,19].

In developing countries such as Ecuador, access to natural products is very low or almost nil due to their higher costs. As the population growth is concentrated in an “urban middle class”, which is pushing the demand for quality food, low in fat, high protein content, and friendly to the environment, the development of an “antimicrobial market”, would be an economic and technological challenge. On the opposite side, the “urban low-income class” lacks the possibility of consuming good quality foods. Their food is mostly purchased from the local open market, mobile vendors, or on the streets. These mobile food stalls are part of the cultural behavior, and the microbiological food safety hazards of these units are rarely evaluated [20]. A preliminary study regarding the bacteriological analysis of 20 natural juices sold out in the open market indicated the presence of several pathogens. Among them, we detected *Citrobacter freundii* and *Staphylococcus* spp. in 80% and 10%, respectively, of the analyzed samples (not published). Although food products of animal origin are considered the main source of the spread of antibiotic resistance, fresh foods of plant origin such as natural juices are becoming a current concern. Besides, susceptibility tests towards different antibiotics showed that the *C. freundii* isolate was a multidrug-resistant strain. Little is known, however, of the extent to which multidrug-resistant bacteria from tropical juices disseminate after ingestion or the precise mechanism for specific bacteria to colonize the human gut. However, the addition of probiotics or their metabolites to reduce the growth of these undesirable microorganisms has barely been investigated. 

In previous studies, we selected three probiotic strains, *L. plantarum* UTNGt2, *L. plantarum* UTNGt21A, and *L. lactis* UTNGt28, exhibiting a high antimicrobial spectrum towards both Gram-positive and Gram-negative bacteria [21,22]. These strains were found to produce peptides that reduce the growth of various commensal microorganisms in vitro and ex vitro. Based on the whole genome sequence analysis, we showed that UTNGt2 and UTNGt21A harbored a plethora of genes participating in antimicrobial activity, with the UTNGt21A strain displaying a complex bacteriocin cluster gene organization and dissimilar gene variants that might contribute to the overall inhibitory capacity [23]. Instead, UTNGt28, a *Lactococcus lactis* species, contains genes encoding for several bacteriocins, such as lactococcin A, lactococcin M, and lacticin [20]. In this study, we evaluated the inhibitory activity of peptide-protein extracts from these bacteriocinogenic strains, alone or in combination against *S. aureus* ATCC1026 and *C. freundii* UTNB3Sm1, to select the optimal inhibitory PCs and further investigate their possible mechanism of action against both target strains by determining the effects on membrane integrity and cell ultrastructural morphology. 

## 2. Results and Discussion

### 2.1. Several PCs Inhibit Both Target S. aureus ATCC1026 and C. freundii UTNB3Sm1 Microorganisms

The inhibitory activity of bacteriocins from lactic acid bacteria against *Staphylococcus* was evaluated earlier [24]. In addition, a bacteriocin from *Pediococcus pentosaceus* showed inhibitory activity against *C. freundii* [25]. Recently, we showed that by combining peptide extracts from different LAB species the inhibitory activity enhanced considerably [23]. As the inhibitory efficiency depends on the target, bacteriocin type and dose applied, each antimicrobial individually or a combination thereof should be evaluated separately. In this study, both targeted *S. aureus* ATCC1026 and *C. freundii* UTNB3Sm1 are multidrug-resistant strains. Thus, peptide-protein extracts from two early characterized bacteriocinogenic *L. plantarum* native strains, UTNGt2 and/or UTNGt21A, in combination with peptide extracts from *L. lactis* UTNGt28, in different doses and co-treated or not with EDTA were evaluated against both resistant strains. The titer (1x MIC equivalent) was estimated at 400 AU/mL for both PC1 and PC3 and 800 AU/mL for PC5 toward *S. aureus*, while the values towards *Citrobacter* were 400 AU/mL, 800 AU/mL, and 1600 AU/mL for PC1, PC3, and PC5 respectively. These results indicated that the inhibitory effect depends on the target, the type of peptide-protein mixture and dose applied, both targets being more susceptible to PC5. The antimicrobial results indicated that out of the 19 tested PCs, the most effective combinations against both target bacteria were PC11 and PC17: consisting of peptide-protein extracts from (UTNGt2 and UTNGt28), and (UTNGt21A with UTNGt28) respectively, with an overloading dose of UTNGt2 and/or UTNGt21A (3 × MIC) versus UTNGt28 (1 × MIC). The addition of EDTA showed a marginal effect on antimicrobial activity (Table 1). EDTA is known as a membrane destabilizing agent and might enhance antimicrobial activity when combined with peptides [21,25]. According to FAO [26], the maximum dose accepted in food is 0.1 mg/mL and is the only food additive approved by EU regulation no. 2008-1333. No inhibitory activity (no inhibition zone) was observed for EDTA alone towards the UTNB3Sm1 strain. In previous research, we showed that by combining UTNGt2 peptide extracts with EDTA, the viability of *E. coli* but not *Salmonella* enhanced considerably [22]. In contrast, by combining the peptide extract from *Weissella cibaria* UTNGt21O strain with EDTA, no increase in the target cell viability was obtained [21]. Likewise, in this study, a marginal effect of EDTA was registered when co-cultivated with peptide-protein extracts independently or a combination thereof towards both pathogens, suggesting a potential synergic effect of the peptide with EDTA on cell lysis or permeabilization depending on the target [27,28]. Overloading of any peptide extracts from *L. plantarum* strains but not *Lactococcus* enhanced the inhibitory effect. However, based on the agar-well diffusion method, we concluded that a combination of peptide extracts from different LAB species results in the inhibition of both resistant strains. 

### 2.2. Overloading of the Peptide-Protein Extracts from UTNGt2 and/or UTNGt21A but Not UTNGt28 Strains Reduced the Target Cell Viability In Vitro

**Figure 1:**
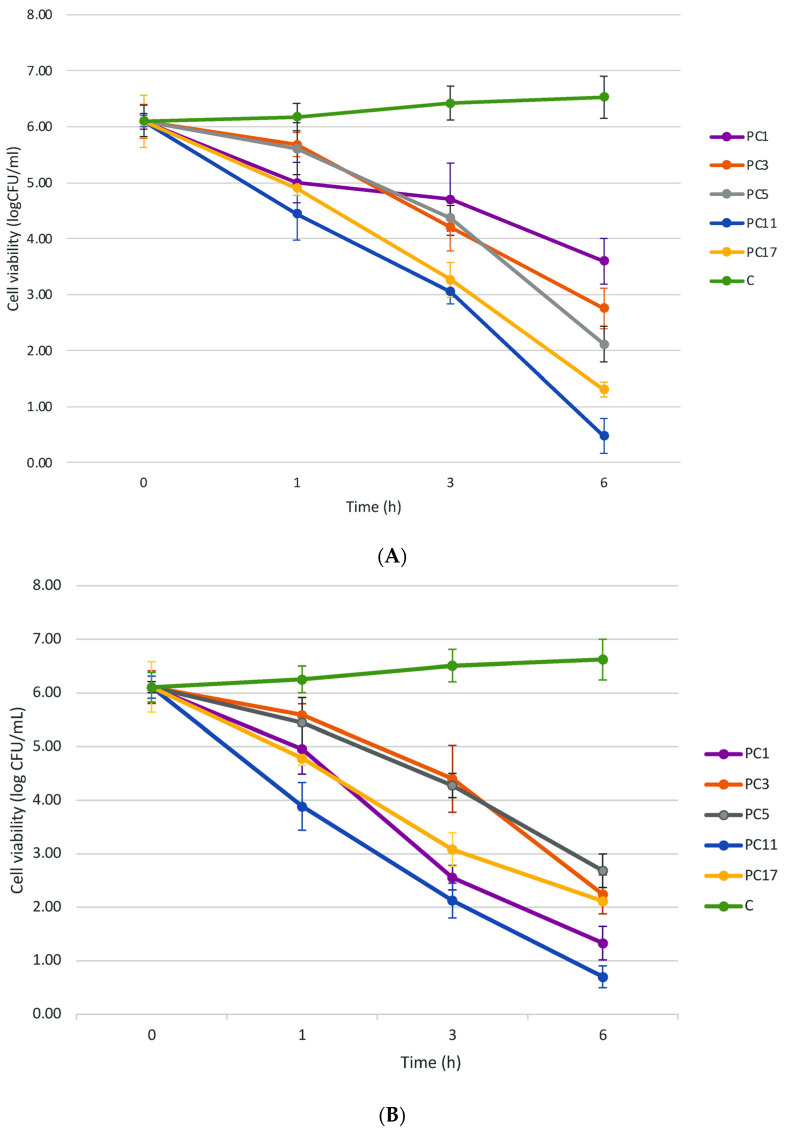

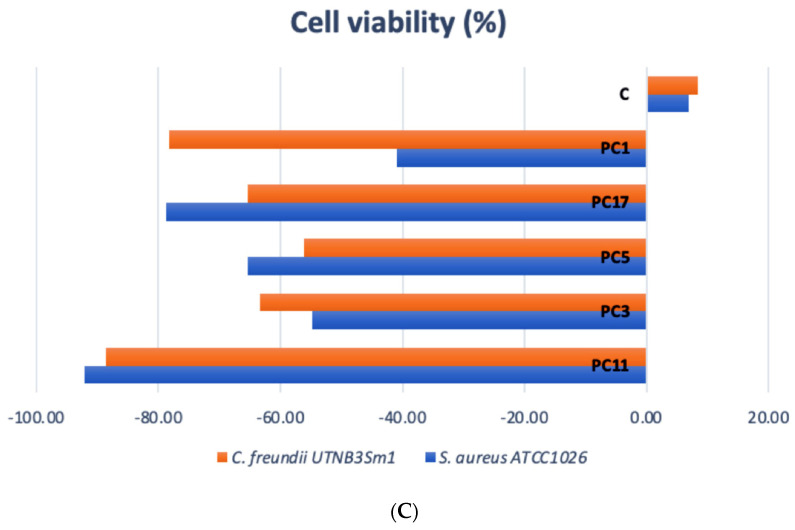
Time-kill curves of (**A**) *S. aureus* ATCC1026; (**B**) *C. freundii* UTNB3Sm1. Error bars represent the standard deviations of three replicates *(n* = 3). (**C**) The percentage (%) of cell viability upon PC treatment; Legend: PC1: 1 × MIC (UTNGt2)PP; PC3: 1 × MIC (UTNGt28)PP; PC5: 1 × MIC (UTNGt21A)PP; PC11: (UTNGt2: UTNGt28)PP (3 × MIC:1 × MIC); PC17: (UTNGt21A: UTNGt28)PP (3 × MIC: 1 × MIC); C1: *S. aureus* ATCC1026 untreated cell culture; C2: *C. freundii* UTNB3Sm1 untreated cell culture; C: untreated cells.

**Figure 2:**
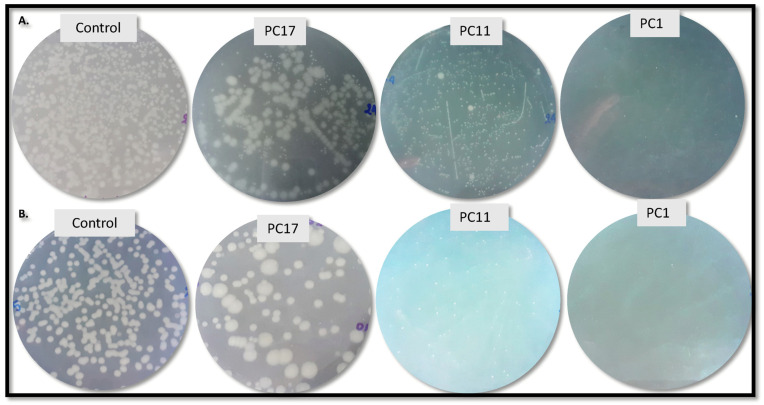
Phenotype of *S. aureus* ATCC1026 (**A**) and *C. freundii* B3Sm1 (**B**) in plate agar upon the treatment with PC1, PC11 and PC17. Control: untreated cells. PC1: 1 × MIC (UTNGt2)PP; PC11: (UTNGt2: UTNGt28)PP (3 × MIC: 1 × MIC); PC17: (UTNGt21A: UTNGt28)PP (3 × MIC: 1 × MIC).

The time-kill curves were determined by exposing *S. aureus* and *Citrobacter* to selected PCs (Figure 1A,B). At its dose, PC11 reduced progressively the cell viability of *Staphylococcus* from 6.10 log CFU/mL (0 h) to 0.48 log CFU/mL (6 h) which corresponds to a 92.18% reduction (Figure 1C). Similarly, PC17 reduced the cell viability from 6.10 log CFU/mL (0 h) to 1.30 log CFU/mL (6 h), which corresponds to a 78.65% reduction. The individual PC1, PC3, and PC5 diminished the cell viability by 40.99%, 54.79%, and 65.35%, respectively (Figure 1C). An increase in bacterial viability was observed in the control (no peptide treated) upon 6 h of incubation. Peculiarly, a phenotype of arrested-growing *S. aureus* cells in agar plates was noted upon treatment with PC11 but not PC17 (Figure 2). The cells showed a transparent appearance and were small compared with the untreated counterpart (Figure 2A). Early research indicated that a small-colony phenotype of *S. aureus* is associated with the lack of thymidine in growth media [29,30]. In another study, this phenotype of cell dormant variants was observed in pathogenic cells resistant to antibiotic treatment [31]. Likewise, *Citrobacter* viability was reduced by 88.5% after 6 h of incubation with PC11 (Figure 1C). The cell concentration reduced progressively from 6.11 log CFU/mL (0 h) to 0.7 log CFU/mL at 6 h, indicating bacterial cell lysis (Figure 1B). The same phenotype of barely visible small cells was observed when treated with PC11 (Figure 2B). We speculated that this phenomenon might be related to the presence of the UTNGt2 peptides in the sample as the treatment with UTNGt2 (PC1) only resulted in a 78.23% reduction (from 6.10 log CFU/mL at 1.33 log CFU/mL). We suggest that these peptide extracts might be sufficient to kill *Citrobacter* in the same manner as PC11 by blocking their growth. We speculate that the peptide mixture accumulation on the cell surface might result in cell lysis as a primary event, while the phenotype of small cells might be a secondary effect of PC interaction with resistant cells. The treatment with PC17 resulted in cell viability reduction by 65.39%, while the control showed an increase in cell viability after 6 h (Figure 1C). Previous research showed the efficiency of bacteriocins from *Bacillus* E50-52 and *Paenibacillus polymyxa* B 602 against several Gram-positive (*S. aureus*) and Gram-negative (*Acinetobacter baumannii*, *C. freundii, E. coli, Proteus*, etc) [32]. Another study showed that bacteriocins PlnEF and PlnJK from *L. plantarum* caused rapid and significant lysis of *S. epidermidis* and induced lysis of liposomes. The PlnEF and PlnJK displayed similar mechanisms by disrupting the bacterial cell membrane [33]. In previous research, we showed that both UTNGt2 and UTNGt21A genomes contain the *pln* locus [23]. However, in this study, the killing effect against multidrug-resistant strains was enhanced by mixing peptide extracts from native *L. plantarum* and *L. lactis* species in a dose and peptide extract type-specific manner. 

### 2.3. PC Treatment Compromised the Cell Membrane Integrity of Target Bacteria

**Figure 3:**
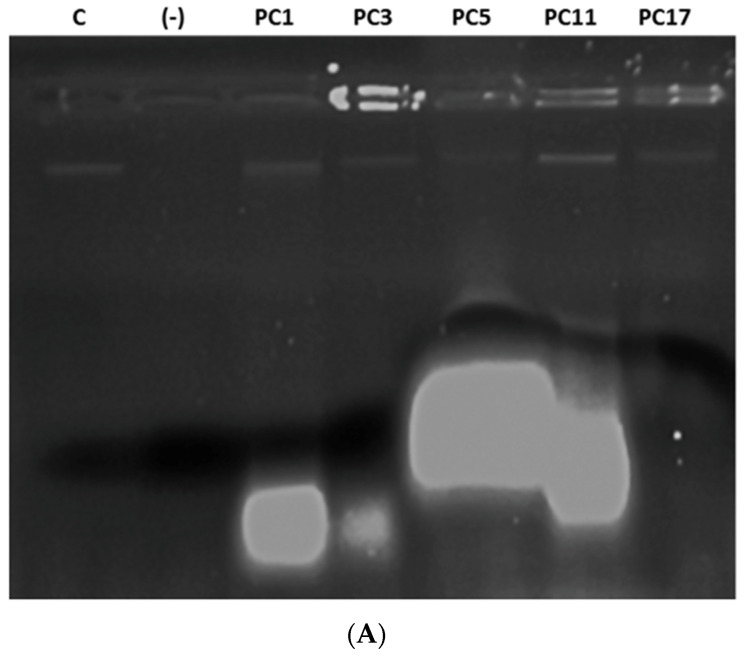

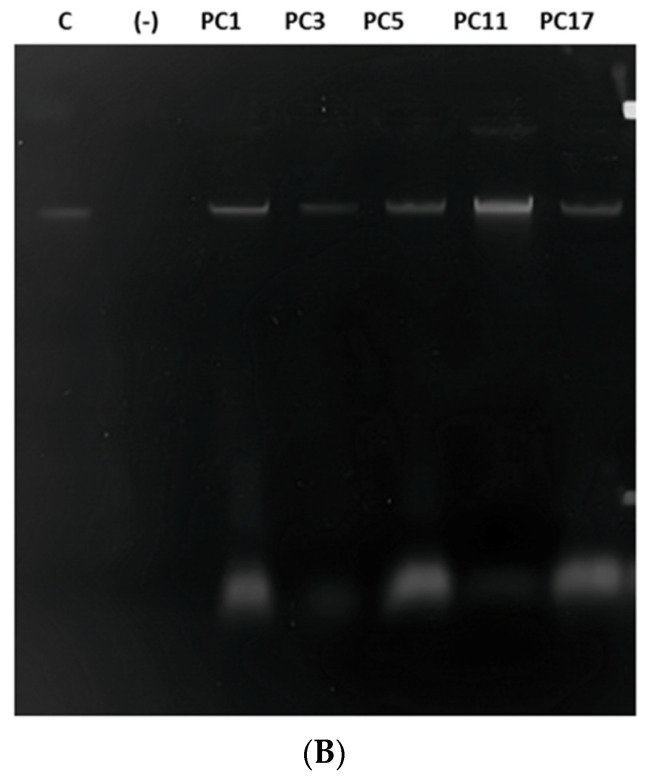
Cell membrane integrity study. Electrophoresis gel showing the presence of DNA/RNA molecules released after the treatment of *S. aureus* ATCC1026 (**A**) and *C. freundii* UTNB3Sm1 (**B**) with PCs. Legend: C: genomic DNA isolated from culture of *S. aureus* and *C. freundii*; (-): negative control (absence of DNA/RNA of untreated target with PCs); PC1: 1 × MIC (UTNGt2)PP; PC3: 1 × MIC (UTNGt28)PP; PC5: 1 × MIC (UTNGt21A)PP; PC11: (UTNGt2: UTNGt28)PP (3 × MIC: 1 × MIC); PC17: (UTNGt21A: UTNGt28)PP (3 × MIC: 1 × MIC).

The cytoplasm of bacteria contains critical biomolecules protected by the plasma membrane and the cell wall [34]. The cell wall of *S. aureus* is thick (15–80 nm), consisting of several layers of peptidoglycan, whereas the Gram-negative *C. freundii*, has a relatively thin (10 nm) cell wall surrounded by an outer membrane. The plasma membrane is the barrier that separates and protects the cytoplasm from the environment [35]. Disorganization of the membrane by external substances can cause loss of the permeability or the integrity of the membrane, inducing cell death [24]. Previous studies have reported that lactobacilli peptides cause severe membrane damage through the disruption of membrane integrity [35]. Bacteriocin F1 from *L. paracasei* subsp. *tolerans* FX-6 disrupts the membrane of *S. aureus* [24]. Therefore, we investigated the effect of PCs on the membrane integrity of both *S. aureus* and *C. freundii* strains. The results showed that the exposure of *S. aureus* cells to each PC caused leakage of DNA and RNA from the cytoplasm (Figure 3A). Although we did not quantify the released nucleic acids upon the PC treatment, a remarkably higher amount of RNA was visible in agarose gel when *S. aureus* cells were treated with PC5 and PC11. At this point, it is difficult to predict why the RNA molecules retard in migration and showed a larger size than observed when treated with other PCs, further analyses are required to explain this phenomenon. The treatment with PC17 resulted in the release of DNA only, suggesting that this combination might cause RNase activity. The electrophoresis profile of *Citrobacter* showed the presence of both DNA and RNA molecules for all PCs (Figure 3B), indicating that either PC from one single strain or PC from different strains disrupts the *Citrobacter* membrane inducing the release of nucleic acids and, eventually, cell death. Thus, we speculated that the loss of membrane integrity upon peptide extract treatment induces the malfunction of the cell permeability barrier and subsequently cell viability loss.

### 2.4. Individual PCs Induced Several Membrane Morphological Changes of the Target Strains

**Figure 4:**
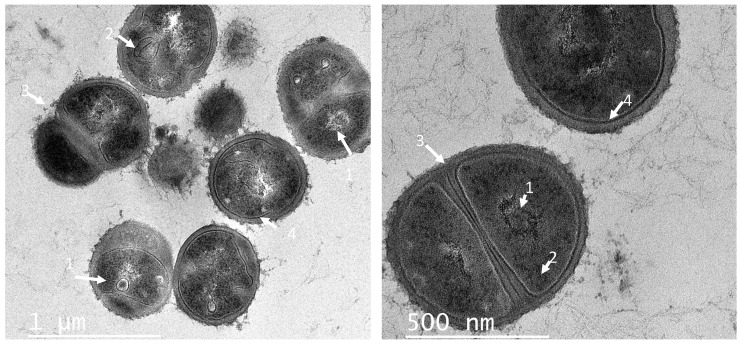
TEM of ultrathin sections of *S. aureus* control (untreated) cells. Legend: 1—nucleoid; 2—mesosome; 3—septum; 4—cell wall. Scale bars correspond to 1 μM and 500 nm.

**Figure 5:**
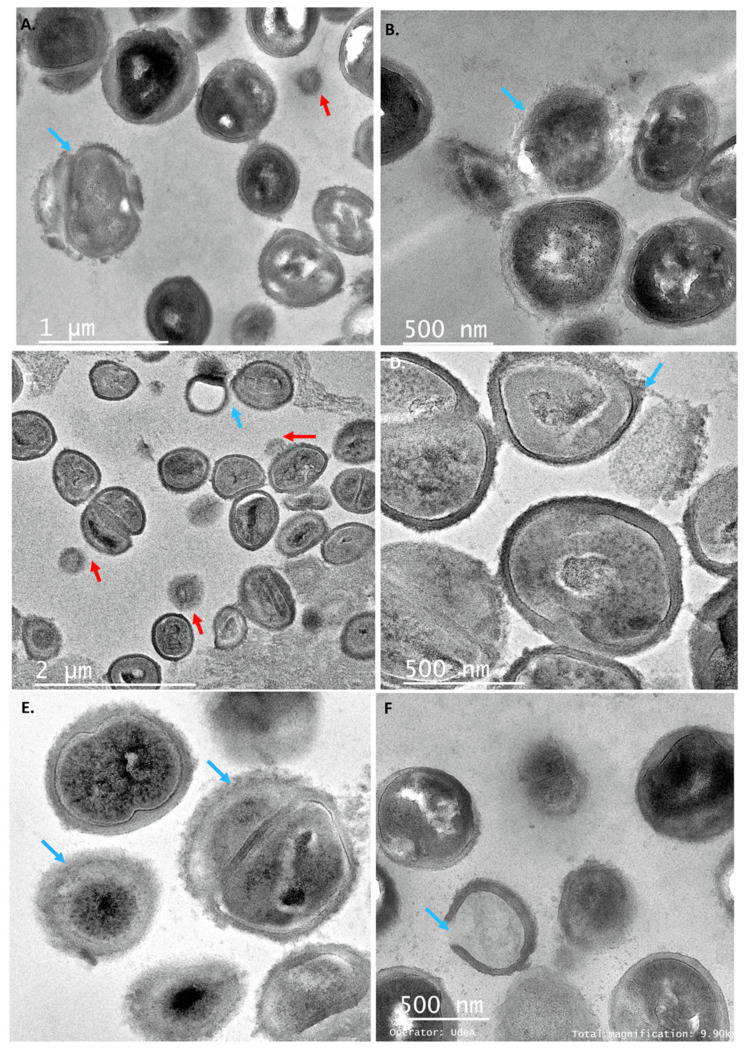
TEM ultrathin sections of *S. aureus* cells: (**A**,**B**) cell incubated with peptide PC1; (**C**,**D**) cells incubated with peptide PC3; (**E**,**F**) cells incubated with peptide PC5, at 1 × MIC for 6 h. The arrows showed different degrees of deformation of cells, condensed cytoplasm, and when the cell wall was damaged; the empty cytoplasm (**C**,**F**) was noticed as cell wall was completely broken. The red arrows showed the membrane blebbing. The blue arrows indicated the cell wall damage and ghost cells. Scale bars correspond to 1, 2 μm, and 500 nm. PC1: 1 × MIC (UTNGt2)PP; PC3: 1 × MIC (UTNGt28)PP; PC5: 1 × MIC (UTNGt21A)PP.

The morphological changes in *Staphylococcus* cells resulting from exposure to PC1, PC3, and PC5 were evaluated by TEM. Untreated cells (Figure 4) exhibited normal round-shaped morphology, with intact cell membranes, and they were grouped in tetrads or showed the formation of septa as an indicator of cell division. When treating *S. aureus* with PCs at its MIC, the cells lost their morphological integrity (Figure 5). The cells showed different degrees of deformation and cytoplasm condensation (Figure 5A,B). Besides, the cell wall was disrupted, while in some cells the empty cytoplasm (Figure 5C,F) was observed due to the leakage of the cell content (ghost cell phenotype). Previously, this phenotype was detected when *Salmonella* cells were treated with UTNGt28 peptides. In this study, such a phenotype was observed upon treatment with either PC3 or PC5. Recent studies using synthetic peptides towards *S. aureus* indicated that the electronic density of the outer border is associated with the presence of teichoic acid [36]. In other studies, plantaricins from *L. plantarum* acted synergistically with antibiotics killing *S. epidermis* [33]. The treatment with individual peptides, PlnA and PlnE resulted in the leakage of the cell content, as well as the exposure to PlnEF and PlnJk resulting in a significant alteration of *S. epidermis* ultrastructure and severe cell damage. However, PC1 and PC5 extracted from plantaricin-producing strains, caused cell wall damage as observed in the TEM section (Figure 5A,E). Besides, the aspect of “fried egg”, and cell wall thickness with a dense outer border was observed when treated with PC3 (Figure 5D). Furthermore, PC3 induced the membrane blebbing of *S. aureus*, indicating that these peptides actuate at the membrane level. These phenomena were detected early in *E. coli* [36] and *S. aureus* [37,38] upon treatment with different antibiotics. An early study on multidrug-resistant *S. aureus* cells showed the fried egg phenotype as the effect of a thymidine-depleted medium [29]. 

**Figure 6:**
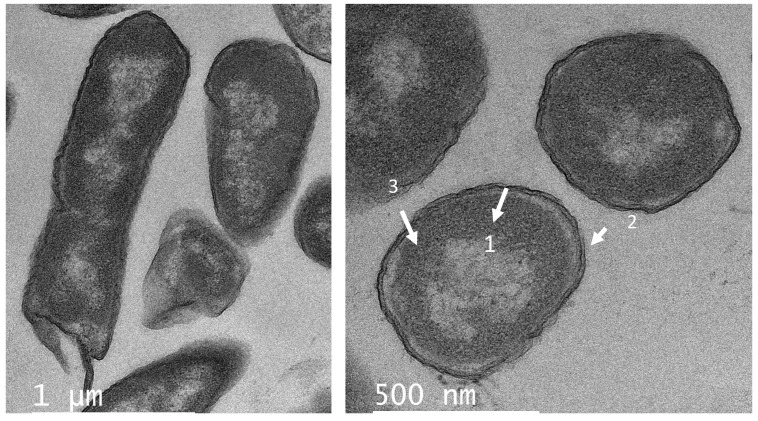
TEM of ultrathin sections of *C. freundii* UTNB3Sm1 control (untreated) cells. Legend: 1—nucleoid; 2—cell wall; 3—cytoplasm. Scale bars correspond to 1 uM and 500 nm.

**Figure 7:**
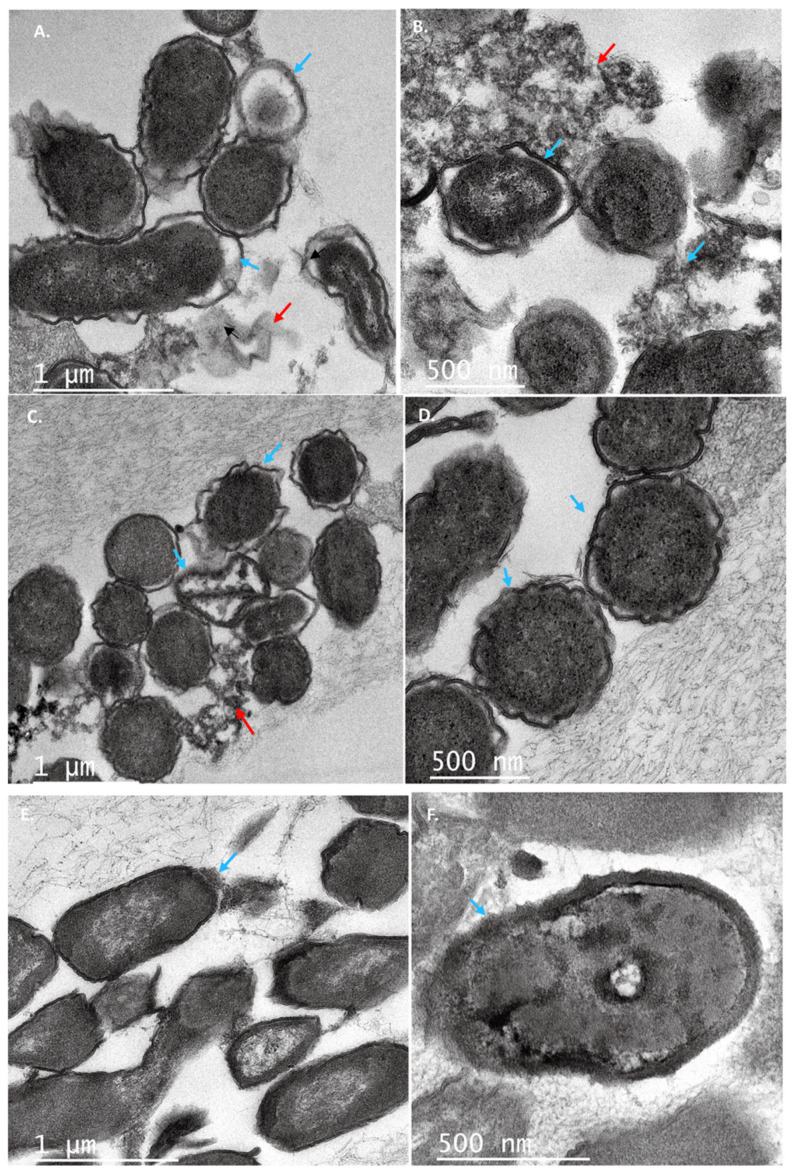
TEM ultrathin sections of *C. freundii* UTNB3Sm1 cells: (**A**,**B**) cell incubated with peptide PC1; (**C**,**D**) cells incubated with peptide PC3; (**E**,**F**) cells incubated with peptide PC5 at 1 × MIC for 6 h. The arrows showed different degrees of deformation of cells (**A**–**F**), leakage of cytoplasm (**B**,**C**,**E**), detached cell membrane (**A**–**C**), undulated cell wall (**A**–**D**), condensed cytoplasm, and ghost cells (**A**); the red arrow indicates the cell debris while the blue arrow indicates the cell morphological modifications. Scale bars correspond to 1 μm and 500 nm. PC1: 1 × MIC (UTNGt2)PP; PC3: 1 × MIC (UTNGt28)PP; PC5: 1 × MIC (UTNGt21A)PP.

Likewise, several changes in the cell shape of *C. freundii* UTNB3Sm1 were observed by TEM. Figure 6 depicts the ultrathin section of control cells showing an intact cell wall. Different degrees of cell deformation (Figure 7A–F), leakage of cytoplasm (Figure 7B,C,E), detached cell membrane (Figure 7A–C), deformed cells with undulated cell wall (Figure 7A–D), condensed cytoplasm, and ghost cells (Figure 7A) were noted after the individual peptide treatment. The cell wall showed a dense outer border when treated with PC5 (Figure 7D). The morphological change of *Citrobacter* upon treatment with peptides from LAB was not well investigated as we were unable to find a publication reporting these types of cell deformations. We believed that these impressive cell shape changes of both Gram-positive and Gram-negative bacteria upon treatment with peptide extracts from native lactic acid bacteria will help with further development of an efficient antimicrobial agent that will overcome the problem of foodborne multidrug-resistant microorganisms. 

### 2.5. SEM Analysis Revealed Different Cell Shape Changes of Both Target Strains upon PC Treatment

**Figure 8:**
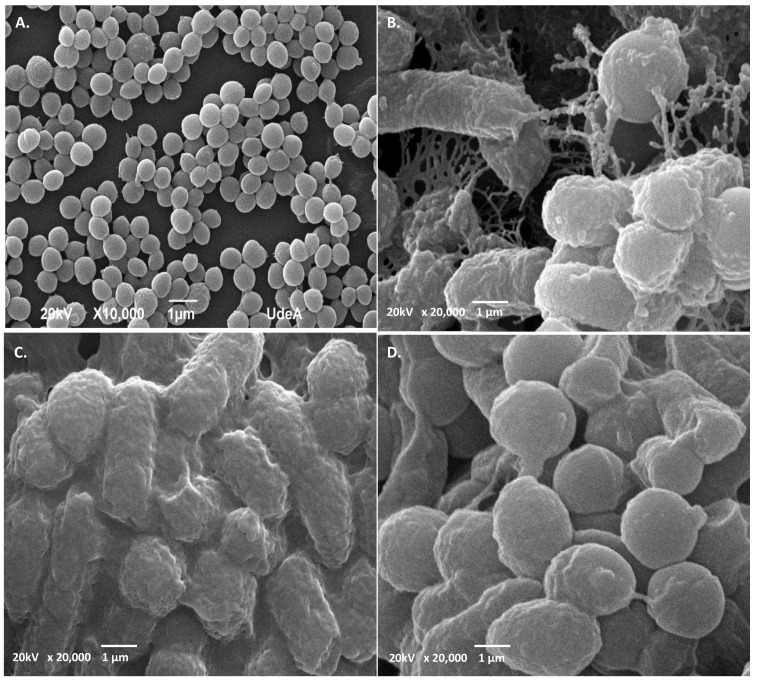
SEM of *S. aureus* ATCC1026. (**A**) control untreated cells; (**B**–**D**) cells incubated with PC1, PC3, PC5 at 1 × MIC for 6 h. The arrows showed different degrees of deformation of cells, wrinkled cells, and debris. Scale bars correspond to 1 μm.

**Figure 9:**
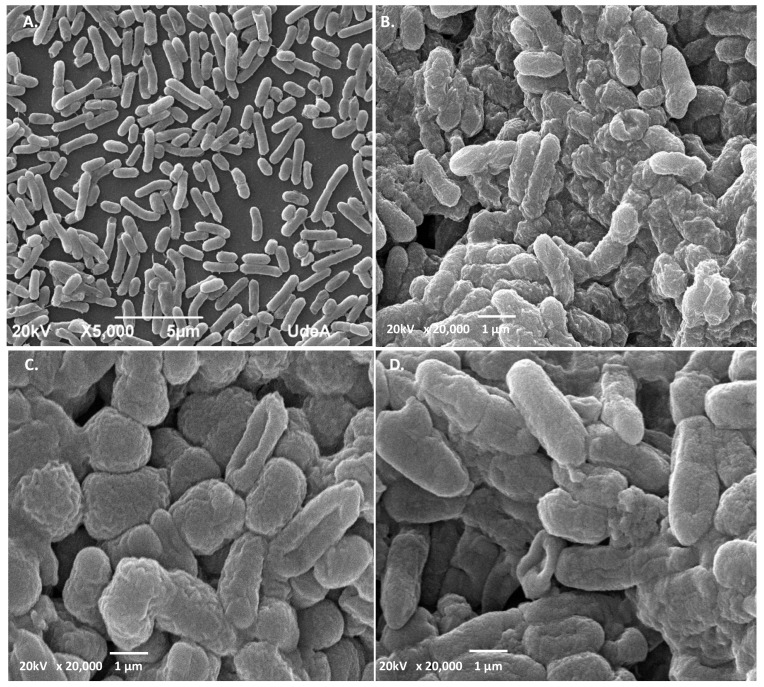
SEM of *C. freundii* UTNB3Sm1. (**A**) control untreated cells; (**B**–**D**) cells incubated with PC1, PC3, PC5 at 1 × MIC for 6 h. The arrows showed different degrees of deformation of cells, wrinkled cells. Scale bars correspond to 1 μm.

SEM micrograph analysis of untreated *S. aureus* ATCC1026 indicated that the cells were intact with smooth surface and cocci shape (Figure 8A), while treatment with PC1, PC3, and PC5 resulted in visible ultrastructural modifications (Figure 8B–D). Thus, the cells showed a wrinkled shape, distorted cell morphology, covered by debris when treated with PC1 (Figure 8B). When treated with PC3, the cells showed different degrees of deformation, septum disappearance, and visible aspect of sticky cells (Figure 8C), while the treatment with PC5 showed wrinkled cells with blebs (visible protrusions of the outer membrane) (Figure 8D). The ultrastructural alteration of *Staphylococcus aureus* was shown when the cells were treated with a cyclic ASP-1 peptide isolated from *Bacillus subtilis* [39]. Although blebs can occur during normal bacterial growth, their increase was associated with certain antibiotics and biocide treatments [40]. In the case of *C. freundii*, SEM micrographs showed intact, larger bacilli cells (Figure 9A) in control, and the presence of wrinkled, deformed with ovoid shape (Figure 9B–D), upon PC treatment. The ovoid cells might form when the rod-shaped bacteria decrease in length due to the inhibition of lateral wall peptidoglycan synthesis [41]. To our knowledge, these types of modifications were not previously shown on *C. freundii* treated with AMPs from lactic acid bacteria. As *Citrobacter freundii* strains were found resistant to several antibiotics [41], their growth and harmless colonization in humans should be evaluated using novel AMPs including peptides from LAB species. In a previous Tricine-SDS-PAGE analysis, we showed that UTNGt2 has four larger products of 22, 32, 35, and 55 kDa, suggesting that more than one molecule might be responsible for the overall inhibitory activity toward different target strains [22]. Furthermore, UTNGt21A yielded several bands of 10, 25, 27, 55, and 75 kDa. The UTNGt28 strain produces a single molecule of 15 kDa [23]. Being larger than peptides these mixture of molecules might be considered “wall breakers”, explaining their activity against multidrug resistant strains, but further analysis is required to detect all the molecules (small and larger) in the extract. According to recent reports, enterolisyn A bacteriocin belongs to this category, acting at the cell wall level inducing the cleavage of the peptide bridge between L-alanine and D-glutamic acid which might explain the antimicrobial effect [19]. From the genome analysis, the UTNGt21A strain harbor a gene similar to enterolisyn A (data not published) along with other bacteriocin encoding genes that might explain its inhibitory activity and distinct ultrastructural modifications than UTNGt2. Overall, we speculate that the peptides might accumulate on the cell surface forming aggregates that might “attack” the bacterial membrane inducing damage or alterations, followed by cell integrity loss with the release of nucleic acids from the cytoplasm and finally cell death. The ultrastructural cell shape changes are secondary events induced by the antimicrobial agents that might share a similar mechanism of action.

## 3. Materials and Methods

### 3.1. Microorganisms

*L. plantarum* UTNGt2 (GenBank Accession No.KY041688.1), *L. plantarum* UTNGt21A (GenBank genome assembly BioProject PRIJNA740042), and *L. lactis* strain UTNGt28 (GenBank accession no. MG675576.1) were previously isolated from wild fruits of Amazon Forest following the procedure as described [20,22,23]. Stocks of these strains were maintained at −80 °C in 20% glycerol (*v*/*v*). Fresh cultures were obtained by cultivation on MRS (Man, Rogosa, and Sharpe) agar (Difco, USA) at 37 °C before use. The target indicator bacterium *S. aureus* ATCC1026 (methicillin-resistant) was obtained from American Type Culture Collection, and *C. freundii* strain UTNB3Sm1 was isolated from tropical juice (laboratory) were grown in BHI broth (Brain Heart Infusion, Merck Millipore, MA, USA) before use. The identification of *C. freundii* species was done by standard 16 S rRNA sequencing and the antibiotic susceptibility was performed by disk diffusion method and E-test following the standard protocols (Table S1) [42]. 

### 3.2. Peptide-Protein Extracts Preparation

The partially purified peptide extract preparation (PP) and MIC (minimum inhibitory concentration) values were determined as previously described [21]. In brief, CFS (cell-free supernatant) was extracted by centrifugation at 13,000× *g* for 30 min (4 °C) of the 24 h LAB culture, followed by filtration using 0.22 µm porosity syringe filter (#STF020025H, Chemlab Group, Washington, DC, USA). To obtain PP, ammonium sulfate at 80% saturation was added to the CFS extract followed by overnight incubation with refrigeration without stirring and centrifuged at 8000× *g* for 30 min and 4 °C. The PPs were recovered in 25 mM ammonium acetate (pH 6.5), desalted by using a midi dialysis kit (cat # PURD10005-1KT, Sigma-Aldrich Co. LLC, Saint Louis, MO, USA), was pre-equilibrated with phosphate buffer (pH 7.0), and stored at (−) 20 °C before use. The titer was defined as 2^n^, where n is the reciprocal of the highest dilution that resulted in inhibition of the indicator strain as previously described [43]. The AU of antimicrobial activity per milliliter was calculated as 2^n^ × 1000 µL/10 µL (10 µL was the sample volume that was used in the agar well assay). The plates were incubated for 48 h at 37 °C and checked for the presence of 2 mm inhibition zones or larger. The PP with a determined concentration (ranged from 200 to 9800 AU/mL) was added independently into broth tubes containing the indicator bacteria, incubated for 24 h at 37 °C followed by plate agar to determine the minimum inhibitory concentration (MIC) that reduced the bacterial growth by 90% [38]. 

### 3.3. Establish the Combination of the Peptide-Protein Extracts (PCs) and Antimicrobial Activity Evaluation

The PCs were obtained as follows: (1) PC1: 1 × MIC (UTNGt2)PP; (2) PC2: 1 × MIC (UTNGt2)PP + EDTA (ethylenediaminetetraacetic acid, 0.1 mg/mL); (3) PC3: 1 × MIC (UTNGt28)PP; (4) PC4: 1 × MIC (UTNGt28)PP + EDTA (0.1 mg/mL); (5) PC5: 1 × MIC (UTNGt21A)PP; (6) PC6: 1 × MIC (UTNGt21A)PP + EDTA (0.1 mg/mL); (7) PC7: (UTNGt2: UTNGt28)PP (1 × MIC: 1 × MIC, *v*/*v*); (8) PC8: (UTNGt2: UTNGt28)PP (1 × MIC: 1 × MIC) + EDTA (0.1 mg/mL); (9) PC9: (UTNGt2: UTNGt28)PP (1 × MIC: 3 × MIC); (10) PC10: (UTNGt2: UTNGt28)PP (1 × MIC: 3 × MIC) + EDTA (0.1 mg/mL); (11) PC11: (UTNGt2: UTNGt28)PP (3 × MIC: 1 × MIC); (12) PC12: (UTNGt2: UTNGt28)PP (3 × MIC: 1 × MIC) + EDTA (0.1 mg/mL); (13) PC13: (UTNGt21A: UTNGt28)PP (1 × MIC: 1 × MIC); (14) PC14: (UTNGt21A: UTNGt28)PP (1x MIC:1 × MIC) + EDTA (0.1 mg/mL); (15) PC15: (UTNGt21A: UTNGt28)PP (1 × MIC: 3 × MIC); (16) PC16: (UTNGt21A: UTNGt28)PP (1 × MIC: 3 × MIC,) + EDTA (0.1 mg/mL); (17) PC17: (UTNGt21A: UTNGt28)PP (3 × MIC: 1 × MIC,); (18) PC18: (UTNGt21A: UTNGt28)PP (3 × MIC: 1 × MIC) + EDTA (0.1 mg/mL); (19) PC19: EDTA (0.1 mg/mL). To screen for PC that inhibits both target strains in vitro, the agar-well diffusion method was used as previously described [21]. The indicator strain (100 μL) grown in broth BHI medium (7 log CFU/mL) was mixed with 3.5 mL of soft MRS agar (0.75%), overlaid on Muller–Hilton agar plates, and incubated at 37 °C for 2 h. Each PC (100 μL) was transferred onto wells (6 mm) on overlaid agar, incubated at 37 °C, and subsequently examined for inhibition zones at 48 h. All experiments were performed in triplicate. The results were expressed as mean ± standard deviation.

### 3.4. Time-Killing Assay of the Selected PCs

To evaluate the effect of the selected PCs showing the highest antimicrobial activity according to the results from Section 3.3, as a function of time, we carried out time-kill experiments. Overnight, *Staphylococcus* and *C. freundii* cultures (1 × 10^6^ CFU/mL) were inoculated independently with each selected PC at the 1 × MIC concentration and incubated at 37 °C. As a control, *S. aureus* and *Citrobacter* untreated cell cultures were used. The cell viability was determined at different time intervals (0, 1, 3, and 6 h) using the plate-agar method (BD Difco plate count agar, Fisher Scientific Co. LLC, Hampton, NH, USA). The results were analyzed by determining the percentage of reduction calculated as the difference between log10 (CFU) of the untreated cells (no peptides) and the treated cells (peptide added or a combination thereof). The cell reduction of >60% was considered the most significant (*p* < 0.05) [44]. All experiments were performed in triplicate. The results were expressed as mean ± standard deviation.

### 3.5. Leakage of Aromatic Molecules Assay

The effect of PC on *S. aureus* ATCC1026 and *C. freundii* UTNB3Sm1 cell integrity was performed as described [23]. In brief, overnight bacterial suspensions of each target (1 × 10^5^ CFU/mL) grown in BHI broth (Brain Heart Infusion, Merck Millipore, MA, USA) were washed twice with 1 × PBS (phosphate-buffered saline, pH 7.5) and treated independently with the selected PC for 6 h at 37 °C. Bacterial cell culture without peptide treatment was used as the control. The release of DNA/RNA molecules was detected by electrophoresis in a 1% agarose gel with ethidium bromide, running in 1 × TBE (Tris-borate, EDTA, pH 8.0) buffer (Sigma-Aldrich Co. LLC, Saint Louis, MO, USA) after extraction with chloroform (1:1, *v*/*v*), and precipitated with isopropanol and ammonium acetate (3 M). 

### 3.6. Transmission Electron Microscope (TEM) Examination of Target Cells Treated with PCs

For both TEM and SEM analysis, the exponential phase *of S. aureus* ATCC1026 and *C. freundii* UTNB3sm1 cells at the concentration 1 × 10^6^ CFU/mL were treated independently with 1 × MIC of PC1, PC3, and PC5 for 6 h at 37 °C following the procedure as previously described [20]. Ultrathin sections were prepared and coated on copper grids and stained with uranyl acetate (Sigma-Aldrich Co. LLC, Saint Louis, MO, USA) and lead citrate (Sigma-Aldrich Co. LLC, Saint Louis, MO, USA). The grids (10 random sections per treatment) were examined using the Tecnai G2 F20 transmission electron microscope (FEI Company, Hillsboro, OR, USA).

### 3.7. Scanning Electron Microscope (SEM) Examination of Target Cells Treated with PCs

For SEM analysis, the PCs treated, and untreated bacteria were resuspended in 1 × PBS and subsequently dried at room temperature and fixed with 2.5% glutaraldehyde overnight at 4 °C following a protocol developed by the Laboratory of Microscopy (University of Antioquia, Colombia). The samples were washed 3 times with phosphate buffer for 5 min and the final wash was done with distilled water. The samples were subsequently dehydrated at room temperature with increasing concentrations of ethanol (50, 75, 95, and 100%), 15 min each in the CPD (critical point dryer) of the samples. The samples were fixed on graphite tape and a thin coating of gold of approx. 24.5 nm was applied to each sample using a DENTON VACUUM Desk IV equipment (DENTON VACUUM, Austin, TX, USA) and subsequently analyzed in a high vacuum scanning electron microscope to obtain high-resolution images. The secondary electron detector was used to evaluate the morphology and topography of the samples. The samples were examined using JSM-6490 LV Scanning Electronic Microscopy equipment (JEOL, JSM, MA, USA). 

## 4. Conclusions

The present study showed the efficacy of peptide-protein extracts and a combination thereof from two *L. plantarum* strains and one *L. lactis*, to inhibit *S. aureus* ATCC1026 and *C. freundii* UTNB3Sm1. Time-killing assay revealed that the selected PCs diminished the cell viability of both target strains by more than 75% within 6 h. These combinations likely caused bacterial cell death by compromising cell membrane integrity and ultrastructural modifications. The leakage of aromatic molecules was detected in both targets as the effect of peptide membrane destabilization and loss of cell integrity. Several membrane shape modifications were detected in both target strains after PC treatment, indicating that the combinations of peptide-protein extracts might “attack” the bacterial membrane, inducing damage or membrane alterations, followed by the cell integrity loss with the release of nucleic acids from the cytoplasm and finally cell death. To the best of our knowledge, this is the first study showing ultrastructure modification of multidrug-resistant *C. freundii* strain. However, these combinations act as powerful antimicrobials against multidrug-resistant foodborne pathogens through various simultaneously molecular events that compromise cell growth. We shall further characterize the bacterial extracts’ composition as other active molecules might contribute to the overall antimicrobial effect. Further action for testing the efficacy of these peptide-protein combinations ex vitro is underway. However, this research will contribute to the food sector to adjust and take the necessary additional steps for the food safety practices required to reduce the threat of multidrug-resistant bacteria by using natural substances such as LAB-bacteriocins and a combination thereof. 

## Figures and Tables

**Table 1:** The antimicrobial activity of PCs towards *S. aureus* ATCC1026 and *C. freundii* B3Sm1.

Code	Combination/Concentration	Inhibition Zone (mm) *	
		*S. aureus* ATCC1026	*C. freundii* UTNB3Sm1
PC1	UTNGt2 (1 × MIC)	9.17 ± 0.29	11.03 ± 0.06
PC2	UTNGt2 (1 × MIC) + EDTA (0.1 mg/mL)	9.17 ± 0.29	9.33 ± 0.58
PC3	UTNGt28 (1 × MIC)	9.67 ± 0.58	9.17 ± 0.29
PC4	UTNGt28 (1 × MIC) + EDTA (0.1 mg/mL)	9.67 ± 0.58	9.33 ± 0.58
PC5	UTNGt21A (1 × MIC)	9.10 ± 0.17	9.33 ± 0.58
PC6	UTNGt21A (1 × MIC) + EDTA (0.1 mg/mL)	9.10 ± 0.17	9.10 ± 0.17
PC7	(UTNGt2: UTNGt28) (1 × MIC: 1 × MIC)	9.10 ± 0.17	10.17 ± 0.29
PC8	(UTNGt2: UTNGt28) (1 × MIC: 1 × MIC) + EDTA (0.1 mg/mL)	9.33 ± 0.58	10.17 ± 0.29
PC9	(UTNGt2: UTNGt28) (1 × MIC: 3 × MIC)	9.10 ± 0.17	9.67 ± 0.58
PC10	(UTNGt2: UTNGt28) (1 × MIC: 3 × MIC) + EDTA (0.1 mg/mL)	9.10 ± 0.17	9.10 ± 0.17
PC11	(UTNGt2: UTNGt28) (3 × MIC: 1 × MIC)	12.17 ± 0.29	13.67 ± 0.58
PC12	(UTNGt2: UTNGt28) (3 × MIC: 1 × MIC) + EDTA (0.1 mg/mL)	9.10 ± 0.17	10.17 ± 0.29
PC13	(UTNGt21A: UTNGt28) (1 × MIC: 1 × MIC)	9.10 ± 0.17	9.10 ± 0.17
PC14	(UTNGt21A: UTNGt28) (1 × MIC: 1 × MIC) + EDTA (0.1 mg/mL)	9.10 ± 0.17	9.10 ± 0.17
PC15	(UTNGt21A: UTNGt28) (1 × MIC: 3 × MIC)	9.67 ± 0.58	9.10 ± 0.17
PC16	(UTNGt21A: UTNGt28) (1 × MIC: 3 × MIC) + EDTA (0.1 mg/mL)	9.67 ± 0.58	9.33 ± 0.58
PC17	(UTNGt21A: UTNGt28) (3 × MIC: 1 × MIC)	11.67 ± 0.58	11.10 ± 0.17
PC18	(UTNGt21A: UTNGt28) (3 × MIC: 1 × MIC) + EDTA (0.1 mg/mL)	9.17 ± 0.29	9.17 ± 0.29
PC19	EDTA (0.1 mg/mL)	7.27 ± 0.64	6.10 ± 0.17
MRS broth	Negative control	6.00 ± 0.00	6.00 ± 0.00

* The mean (± standard deviation) of the diameter of the inhibition zone (mm) is shown.

## Data Availability

Not applicable.

## Supplementary Materials

The following are available online at https://www.mdpi.com/article/10.3390/antibiotics11020154/s1, Table S1. Antibiotic susceptibility of the *Citrobacter freundii* B3Sm1.
