# Supplementary material for: Combinations of Peptide-Protein Extracts from Native Probiotics Suppress the Growth of Multidrug-Resistant Staphylococcus aureus and Citrobacter freundii via Membrane Perturbation and Ultrastructural Changes
Source: Antibiotics (Basel). 2022 Jan 25;11(2):154. doi: 10.3390/antibiotics11020154 (PMC8868453; doi:10.3390/antibiotics11020154)
Supplement: Supplementary file 1 [file antibiotics-11-00154-s001.zip › Table S1.pdf]

Table S1. Antibiotic susceptibility of the Citrobacter *freundii* B3Sm1

Antimicrobial agent	Zone diameter of disk (mm)	Interpretation criteria /Susceptibility*	E-test strip MIC (mg/L)	**EFSA cut-off values (mg/L) [EFSA, 2012]
Amoxicillin: clavulanic acid	10	R	32	≥ 32
Ampicillin	6	R	16	≥ 8
Cefotaxime	6	R	8	≥ 4
Gentamycin	7	R	16	≥ 2
Kanamycin	6	R	16	≥ 8
Tetracycline	6	R	32	≥ 8

*The microbiological breakpoints reported by the FEEDAP were used to categorize bacteria as susceptible or resistant. The strains showing a MIC higher than the EFSA breakpoint were considered resistant. Susceptible (S): a bacterial strain is defined as susceptible when it is inhibited at a concentration of a specific antimicrobial equal to or lower than the established cut-off value (S ≤ x mg / L). Resistant (R): a bacterial strain is defined as resistant when it is not inhibited at a concentration of a specific antimicrobial above the established cut-off value (R> x mg / L). ** MIC reference for *E. coli*
